# Exploration of the Roles and Mechanisms Between Tumor-Infiltrating Lymphocytes and Hepatocellular Carcinoma Based on Single-Cell Transcriptomics

**DOI:** 10.1155/ijog/1575734

**Published:** 2025-08-20

**Authors:** Taiyu Shi, Donghao Cheng, Xinyan Du, Jianning Wang, Siyuan Jiang, Liangyu Yang, Fen Chen, Chenxu Gong, Yan Ou, Bangjie Chen, Yiwen Jia

**Affiliations:** ^1^The First Clinical Medical College of Anhui Medical University, Hefei, China; ^2^Department of Gastroenterology, The Third Affiliated Hospital of Anhui Medical University (Hefei First People's Hospital), Hefei, China

**Keywords:** hepatocellular carcinoma, machine learning, postoperative prediction model, tumor-infiltrating lymphocytes

## Abstract

Hepatocellular carcinoma (HCC) remains a major global health challenge, with limited effective treatment options, particularly in advanced-stage patients. The tumor immune microenvironment (TIME) plays a crucial role in HCC progression and treatment response, with tumor-infiltrating lymphocytes (TILs) being key modulators of immune activity. In this study, we investigated the immunosuppressive role of TIL-related genes in NASH-associated HCC (NASH-HCC) and identified their potential as independent prognostic factors. We employed Gene Set Enrichment Analysis (GSEA) and Weighted Gene Coexpression Network Analysis (WGCNA) to explore immune suppression in NASH-HCC and identify TIL-related gene modules. Machine learning approaches were utilized to construct a prognostic model, validated using multiple cohorts from the Gene Expression Omnibus (GEO) and The Cancer Genome Atlas (TCGA). The model's predictive power was assessed using Kaplan–Meier survival analysis and receiver operating characteristic (ROC) curves. Furthermore, single-cell RNA sequencing (scRNA-seq) analysis was performed to examine the role of TIL-related genes in different immune cell populations within TIME. We identified 10 distinct cell types in HCC and demonstrated that T cells exhibited the highest TIL pathway activity, playing a critical role in cellular communication via MIF signaling. Our findings highlight the immunosuppressive nature of TILs in NASH-HCC and provide valuable insights into their prognostic significance, potentially guiding future immunotherapeutic strategies.

## 1. Introduction

According to data released by the National Cancer Center of China, there were 367,700 new cases of primary liver cancer nationwide in 2022, ranking fourth among all newly diagnosed cancers and fifth in incidence rate. Approximately 80%–90% of primary liver cancers are hepatocellular carcinoma (HCC) [1, 2]. Despite recent advancements in early screening and diagnosis of HCC, clinical treatment still faces significant challenges. The heterogeneity of HCC and its complex tumor immune microenvironment (TIME) limit the effectiveness of traditional treatment methods such as surgical resection, radiotherapy, and chemotherapy. This is particularly evident in advanced-stage patients, for whom effective treatment strategies remain lacking [3, 4]. With the approval of immune checkpoint inhibitors (ICIs) (including atezolizumab/bevacizumab, CTLA-4 antibody ipilimumab, and the PD-1 antibody pembrolizumab), the focus of HCC clinical trials has shifted toward immunotherapy [5, 6]. The main reason for tumor immune evasion is the dysfunction of cytotoxic CD8+ T cells and the excessive presence of inhibitory T cells. Therefore, the primary focus of current immunotherapy in target discovery remains on CD8+ T cells and inhibitory T cells [7]. However, compared to the successes achieved in melanoma and lung cancer, there have been relatively few reports of successful immunotherapy strategies for liver cancer [8]. To address this issue, a deeper understanding of the TIME of HCC is required.

Tumor-infiltrating lymphocytes (TILs) were previously considered the hosts of the tumor immune response. TILs consist of various lymphocyte subpopulations, including both innate and adaptive immune cells such as mast cells, macrophages, NK cells, and T lymphocytes. They express multiple specific surface antigens, including Foxp3, CD3, CD4, CD8, CD16, CD20, CD56, CD57, CD68, and CD169 [9]. It is widely recognized that Foxp3, CD3, CD4, and CD8 are associated with T lymphocytes, CD16 with monocytes, CD20 with B lymphocytes, CD56 and CD57 with NK cells, and CD68 and CD169 with macrophages. TILs play a crucial role in the development, treatment, and prognosis of liver cancer. Additionally, the antitumor or protumor effects of TILs are related to the composition of lymphocyte subpopulations within the TIME [10]. T lymphocytes are the predominant cells in liver cancer TILs [11]. Moreover, Foxp3+, CD3+, CD4+, and CD8+ T lymphocytes are among the most extensively studied TIL subgroups [12]. Therefore, further research on TIL-related molecules will help us gain a comprehensive understanding of TIME in HCC and significantly advance first-line immunotherapy.

In this study, we focus on the immunosuppressive role of TIL-related genes in HCC and identify them as independent prognostic factors for HCC. Furthermore, we conducted single-cell analysis to investigate the functions of TIL-related genes in different immune cells within TIME. By examining prognosis, tumor-promoting mechanisms, and TIME, we comprehensively evaluated the relationship between TIL-related genes and HCC from multiple perspectives.

## 2. Materials and Methods

### 2.1. Study Design

**Figure 1:**
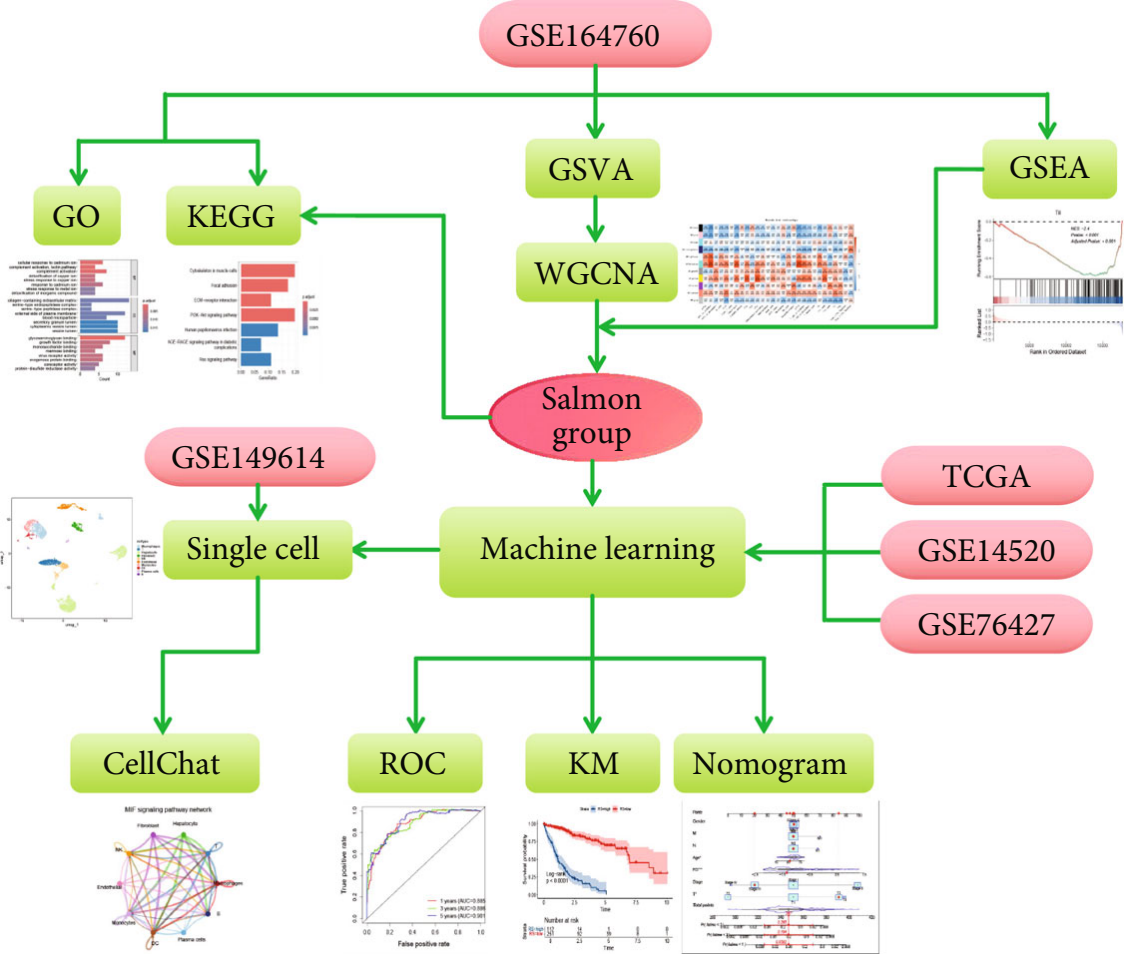
Flowchart of the analysis.

We first conducted Gene Set Enrichment Analysis (GSEA) in NASH-related hepatocellular carcinoma (NASH-HCC) and identified the suppression of TILs. Additionally, Weighted Gene Coexpression Network Analysis (WGCNA) was performed to classify gene modules in the GSE164760. By analyzing the immune function of NASH-HCC and identifying the gene set most strongly correlated with immune activity, we selected genes associated with the TIL pathway from the salmon group. The salmon group is a gene group associated with TIL from WGCNA analysis. Machine learning was employed to identify significant prognostic genes in the salmon module. Receiver operating characteristic (ROC) analysis, Kaplan–Meier (KM) survival analysis, and a nomogram were utilized to evaluate the model's performance. Finally, single-cell analysis was conducted to validate our findings. The complete workflow of our study is illustrated in Figure 1.

### 2.2. Data Sources

Multiple datasets were obtained from the Gene Expression Omnibus (GEO) database and The Cancer Genome Atlas (TCGA) for this study, including GSE164760, GSE14520, GSE76427, TCGA-LIHC, and the single-cell RNA sequencing (scRNA-seq) dataset of HCC patients (GSE149614).

### 2.3. Analysis of RNA-Seq Datasets

We conducted clustering analysis on each sample in the GSE164760 dataset and applied WGCNA [13]. Additionally, we performed immune function analysis and quantified the enrichment levels of immune functions for each sample using the Gene Set Variation Analysis (GSVA) algorithm. GSEA enrichment analysis was performed using the clusterProfiler R package [14]. Additionally, Gene Ontology (GO) and Kyoto Encyclopedia of Genes and Genomes (KEGG) analyses were conducted for functional enrichment assessment.

### 2.4. Establishment of the Prognostic Model

Additionally, a total of 118 algorithmic combinations incorporating 10 machine learning methods were applied to the selected significant prognostic features across the cohorts in our study. These methods included CoxBoost, elastic network (Enet), generalized boosted regression modeling (GBM), least absolute shrinkage and selection operator (Lasso), partial least squares regression for Cox (plsR Cox), random survival forest (RSF), ridge regression, supervised principal components (SuperPC), stepwise Cox regression, and survival support vector machine (survival-SVM) [15, 16]. Moreover, the average concordance index (C-index) of each algorithmic combination across the entire validation set was used as a criterion to assess their performance. The algorithmic combination with the highest average C-index was selected for subsequent model construction.

Besides, we validated the effectiveness of our model in predicting the prognostic characteristics of patients by displaying KM survival curves. Subsequently, we used the R package “survivalROC” to generate time-dependent ROC curves and calculate the area under the curve (AUC) for 1-, 3-, and 5-year survival predictions.

### 2.5. Analysis of scRNA-Seq Datasets

Firstly, we used the Seurat R package to cluster the single-cell dataset GSE149614 and performed basic cell annotation. Cells with a gene count between 200 and 8000, or with a mitochondrial gene ratio of less than 10%, were selected to exclude potential low-quality cells. Harmony was applied to eliminate batch effects among samples, followed by principal component analysis (PCA) for dimensionality reduction and UMAP for visualization. Subsequently, the CellChat R package was used for intercellular communication analysis to identify actively communicating cells, and the UCell R package was employed to calculate the enrichment levels of the TIL pathway. [17, 18].

### 2.6. Cell Culture

The human T-cell leukemia cell line TALL-104 (ATCC CRL-11386) and HCC cell line Hep 3B (ATCC HB-8064) were cultured in RPMI-1640 and DMEM (Gibco) supplemented with 10% fetal bovine serum (FBS) (Gibco), respectively. The MIF-knockout (sgMIF-TALL-104) and negative control (NC-TALL-104) cell lines were generated via the CRISPR-Cas9 system using sgRNA targeting human MIF (sgMIF: 5⁣′-GACGGCTCCATGAACTGGTC-3⁣′) and a nontargeting control sgRNA (5⁣′-GCGAGGTATTCGGCTCCCGT-3⁣′), followed by puromycin selection (2 *μ*g/mL, 7 days). For coculture experiments, Hep 3B cells (3 × 10^3^ cells/well) were seeded in 96-well plates and allowed to adhere for 24 h. Wild-type TALL-104, NC-TALL-104, or sgMIF-TALL-104 cells (mitomycin C-treated, 10 *μ*g/mL, 2 h) were added at a 1:1 ratio (3 × 10^3^ cells/well) for 4 h of coculture. After removing TALL-104 cells by gentle PBS washing, Hep 3B proliferation was assessed using CCK-8 reagent (Dojindo, 10 *μ*L/well, 2 h incubation) at 450 nm (SpectraMax i3x). All experiments were performed in triplicates with three independent replicates. Data were analyzed by one-way ANOVA with Tukey's post hoc test (GraphPad Prism 9.0).

### 2.7. Western Blotting

After coculture with TALL-104 cells (wild-type, Mock, or sgMIF-TALL-104) under the aforementioned conditions, Hep 3B cells in 12-well plates were washed twice with ice-cold PBS and lysed using RIPA buffer (Beyotime, P0013B) supplemented with 1 mM PMSF and protease inhibitor cocktail (Beyotime, P1005) on ice for 30 min. Lysates were centrifuged at 12,000 × g for 15 min at 4°C, and protein concentrations were quantified via BCA assay (Thermo Fisher, 23227). Equal amounts of protein (20 *μ*g per lane) were separated by 10% SDS-PAGE and transferred to PVDF membranes (Millipore, IPFL00010). Membranes were blocked with 5% nonfat milk in TBST for 1 h at room temperature, followed by overnight incubation at 4°C with primary antibodies: anti-ERGIC3 (1:1000, Abcam, ab129179) and anti-*β*-actin (1:5000, Abcam, ab9485) as a loading control. After washing with TBST, membranes were incubated with HRP-conjugated secondary antibodies (1:5000, Cell Signaling Technology, 7074S) for 1 h at room temperature. Protein bands were visualized using ECL substrate (Millipore, WBKLS0500) and quantified by ImageJ software (NIH). Data were normalized to *β*-actin and presented as fold change relative to the control group.

### 2.8. Quantitative Real-Time PCR (qPCR)

Total RNA was extracted from Hep 3B cells (cocultured with TALL-104 variants as described) using TRIzol reagent (Invitrogen, 15596026), followed by DNase I treatment (Thermo Fisher, EN0521) to eliminate genomic DNA contamination. RNA concentration and purity were determined by NanoDrop 2000 (A260/A280 ratio > 1.8). First-strand cDNA was synthesized from 1 *μ*g RNA using the PrimeScript RT Master Mix (Takara, RR036A) with oligo(dT) primers. qPCR was performed on a QuantStudio 5 Real-Time PCR System (Applied Biosystems) with SYBR Green Premix (Takara, RR420A) under the following conditions: 95°C for 30 s, followed by 40 cycles of 95°C for 5 s and 60°C for 30 s. The primers targeting ERGIC3 (Forward: 5⁣′-CAGCTGGACCTCAAGAACCT-3⁣′; Reverse: 5⁣′-GTGCCATCACAGTCACAGGT-3⁣′) and the reference gene *β*-actin (Forward: 5⁣′- CTCCATCCTGGCCTCGCTGT-3⁣′; Reverse: 5⁣′- GCTTCACCTTCACCGTTCC-3⁣′) were designed using Primer-BLAST (NCBI) and validated by melt curve analysis. Gene expression was normalized to *β*-actin and calculated using the 2 − ΔΔCt method. All reactions were performed in triplicate with no-template controls (NTCs) included in each run.

## 3. Result

### 3.1. Immunosuppressive Role of TIL in the HCC

**Figure 2:**
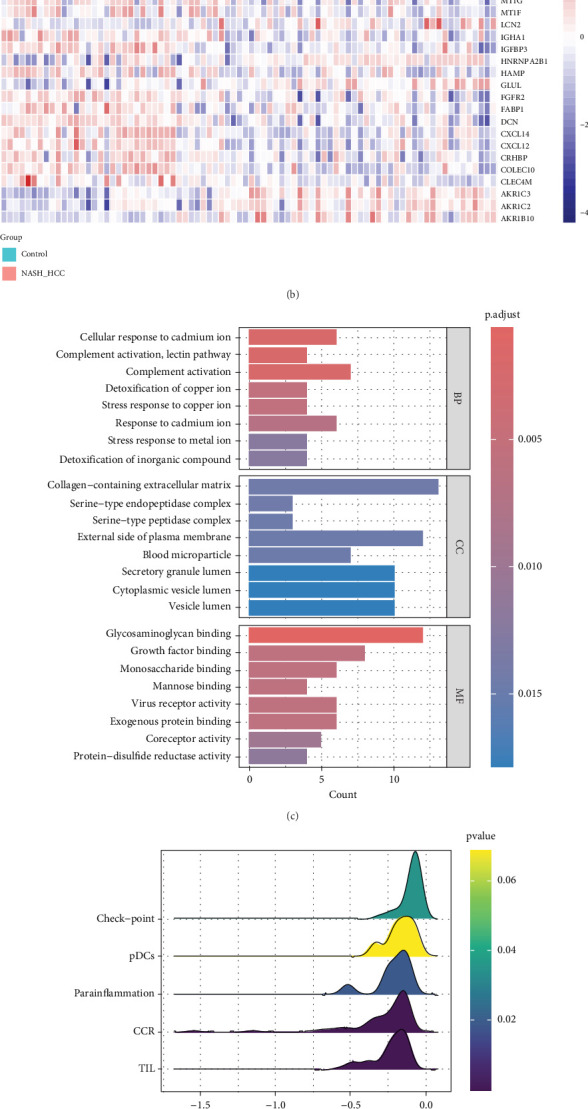
(a) Volcano plot showing differentially expressed genes (DEGs) between groups. (b) Heatmap illustrating DEGs across different groups. (c) GO enrichment analysis depicting the functional pathways significantly enriched in DEGs. (d, e) GSEA analysis results showing the enrichment of specific pathways in the dataset.

In GSE164760, we used a volcano plot to visualize the Top 5 upregulated and downregulated differentially expressed genes (DEGs) compared to normal samples. The DEGs are presented in Figure 2a. Notably, CXCL14, which is downregulated in NASH-HCC, has been shown to be positively associated with the TIL pathway, suggesting its potential role in promoting immune cell infiltration within the tumor microenvironment of oral cavity squamous cell carcinoma [19]. Furthermore, DEGs were visualized using a ComplexHeatmap in Figure 2b. Additionally, GO enrichment analysis revealed that complement activation was enriched in the cellular component (CC) category (Figure 2c). Subsequently, GSEA was conducted to demonstrate the suppression of TIL in NASH-HCC, as illustrated in Figure 2d,e.

### 3.2. Association of TIL-Related Genes With HCC

**Figure 3:**
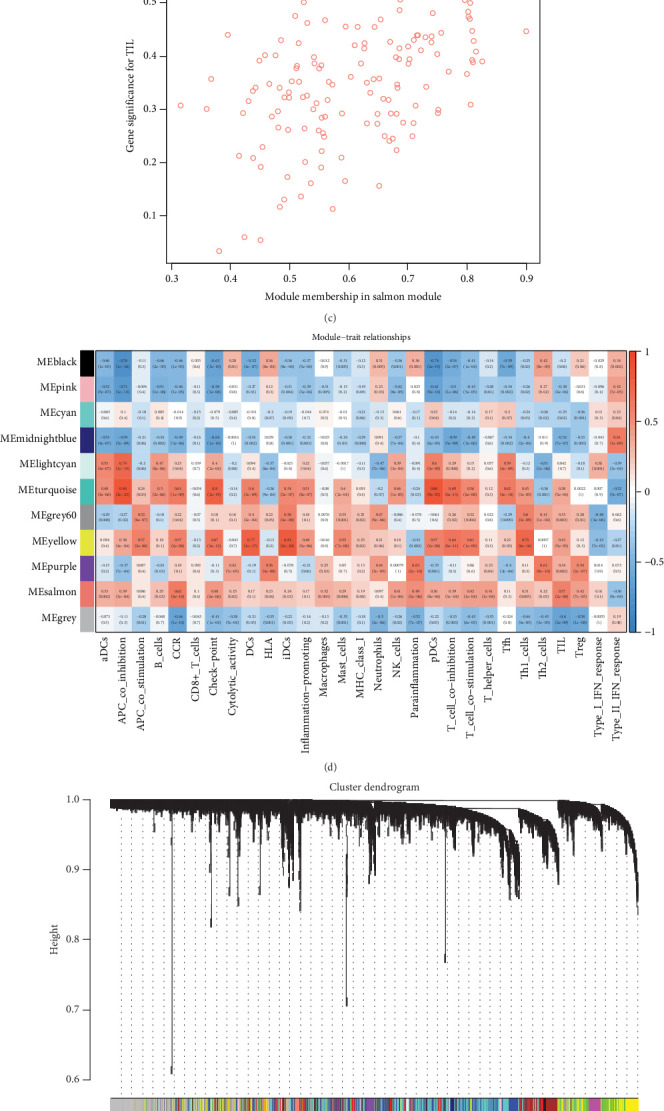
(a, b) Scale-free fitting index analysis for different soft thresholds (power = 9) and mean connectivity for different soft thresholds. (c) Scatterplots of gene significance and the yellow module membership. (d) Heatmap showing module-trait correlations. (e) Gene dendrogram obtained by average linkage hierarchical clustering. The colored row underneath the dendrogram indicates modules as determined by the Dynamic Tree Cut. (f) KEGG enrichment results for the salmon module. (g) GSVA analysis comparing immune function scores across different groups.

The soft thresholding power was set to 0.85 to construct a scale-free network power = 9, as shown in Figures 3a, 3b, and 3e, with a soft threshold value of power = 9 in Supporting Information 3: Table S1. The correlation coefficients between immune function and the module eigengenes of each module were then calculated. The results showed that the salmon module had the highest positive correlation to TIL. The correlation of the module in salmon with gene significance for TIL was 0.43 (*p* = 7.5e^−8^) in Figure 3c. Using WGCNA, we classified all genes into 11 distinct gene modules with different functions, as shown in Figure 3d. The genes in the salmon module were enriched in the KEGG pathway, as illustrated in Figure 3f. Additionally, immune function was assessed using the GSVA algorithm, revealing significant changes in the TIL pathway across different groups (Figure 3g).

### 3.3. Internal Training and External Validation of TIL-Related Gene Signature Prediction Model in Machine Learning

**Figure 4:**
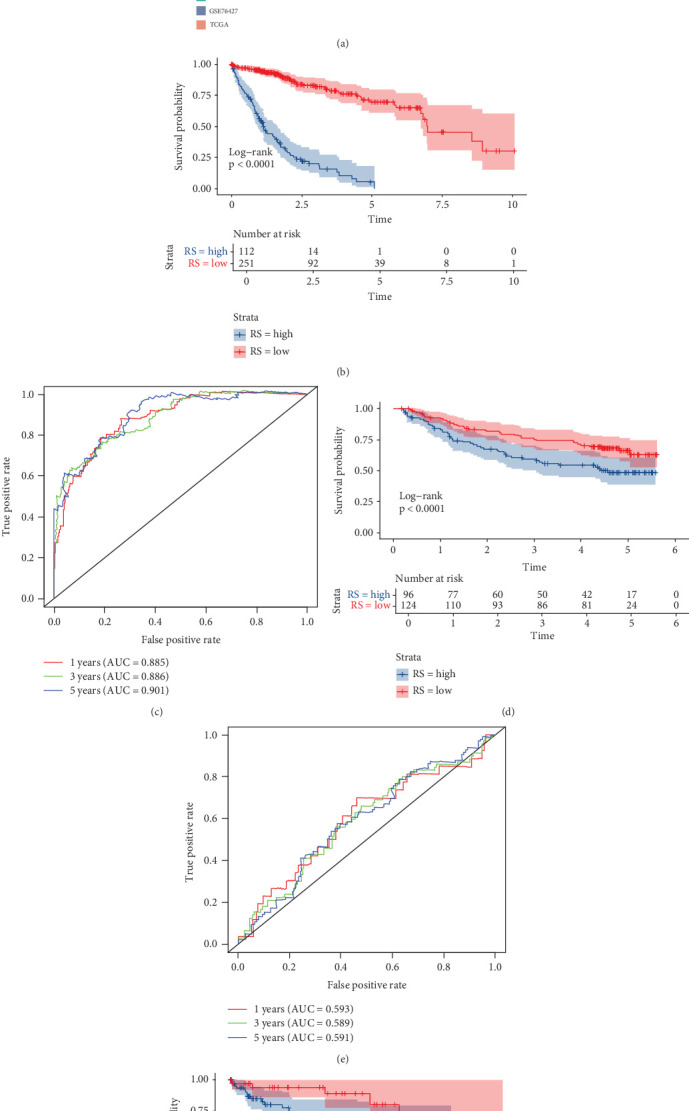
(a) C-index analysis of 118 machine learning models in TCGA, GSE14520, and GSE76427. (b, d, and f) Kaplan–Meier survival analysis for the models in TCGA, GSE14520, and GSE76427, respectively. (c, e, and g) ROC curves showing the area under the curve (AUC) for 1-, 3-, and 5-year survival predictions in TCGA, GSE14520, and GSE76427.

**Figure 5:**
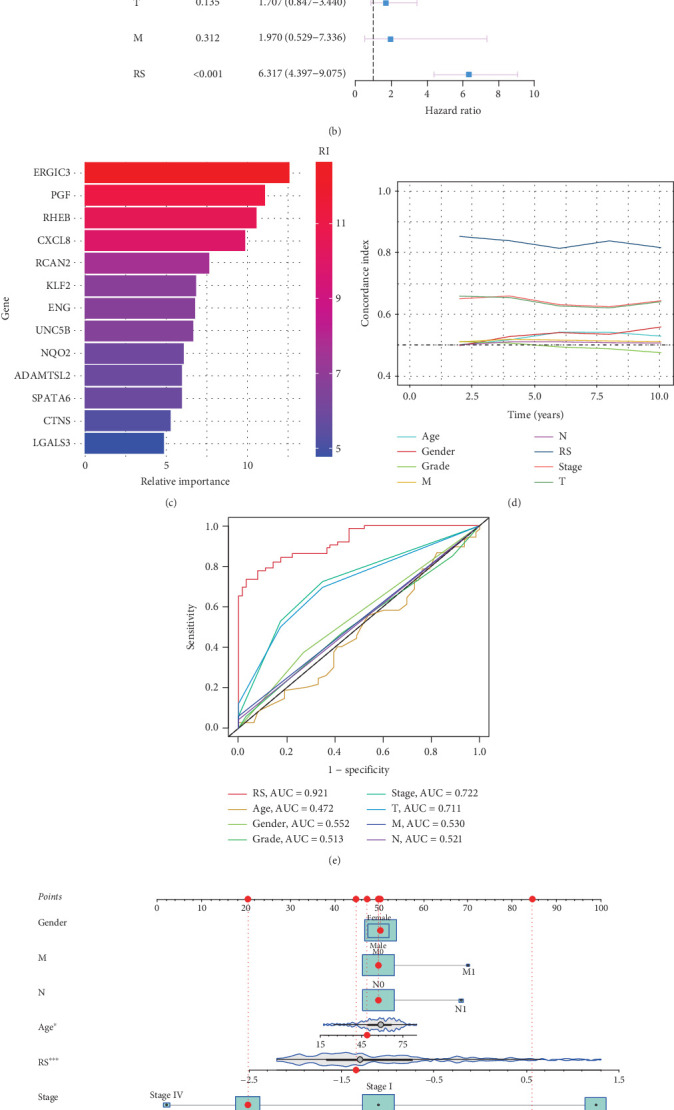
(a, b) Univariate analysis and multivariate analysis for clinical indices along with the model risk score. (c) Relative importance of model genes in the LASSO + GBM combination. (d) C-index for clinical indices along with the model risk score. (e) ROC curves for clinical indices along with the model risk score. (f) Nomogram integrating model risk score and clinical indices to predict the prognosis of LIHC patients.

Using the expression levels of the salmon module, along with survival time and status as input data, a total of 118 algorithmic patterns incorporating 10 machine learning methods were applied within the TCGA-LIHC cohort as the training set, with GSE14520 and GSE76427 as validation datasets. Based on the ranking of the average C-index in the GEO databases, we selected the LASSO + GBM combination, which had the highest average C-index (0.586), for further identification of key prognostic features. Additionally, the prognostic significance of the model features was evaluated using KM curves, with all results demonstrating statistical significance (*p* < 0.05) in Figures 4b, 4d, and 4f. Furthermore, time-dependent ROC curves were generated, showing AUC values for 1-, 3-, and 5-year survival predictions in each cohort, as illustrated in Figures 4c, 4e, and 4g. Subsequently, we expanded our analysis by incorporating clinical indices along with the model risk score to enhance prognostic prediction. Univariate analysis yielded an HR = 6.530 (95% CI: 4.657–9.155, *p* < 0.001), while multivariate analysis showed an HR = 6.317 (95% CI: 4.397–9.075, *p* < 0.001), as presented in Figures 5a,b. The relative importance of model genes was evaluated in Figure 5c. Notably, our model consistently outperformed individual prognostic indices in predictive capability, as demonstrated in Figures 5d, 5e, and 5f. Correlation analysis between the expression of TIL-related genes in the TCGA-LIHC cohort and clinicopathological features was performed in Supporting Information 1: Figure S1. To analyze through which specific signaling pathways the TIL-related genes regulate the occurrence and development process of HCC, we used GO enrichment analysis and GSEA in KEGG pathway to investigate the underlying pathways associated with different expression levels of the TIL-related hub gene ERGIC3 in Supporting Information 2: Figure S2.

### 3.4. Single-Cell Transcriptome of Liver From Patients With HCC

**Figure 6:**
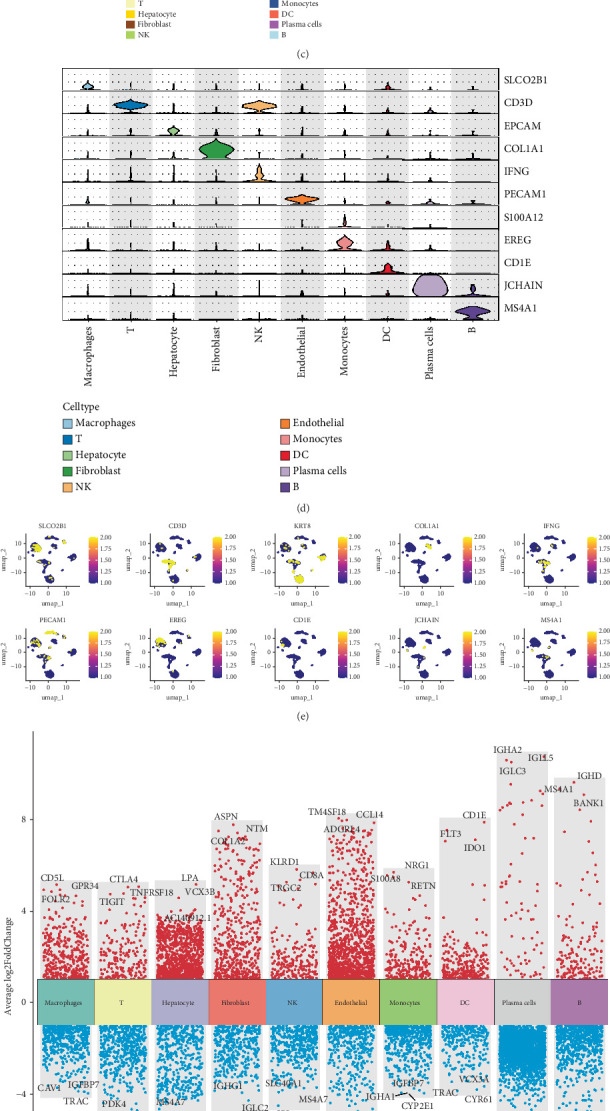
(a) UMAP projection of single-cell cell type. (b) Relative abundance of 10 distinct cell types. (c) Heatmap of gene expression across the 10 distinct cell types. (d) Violin plot displaying marker gene expression for the 10 distinct cell types. (e) UMAP projection highlighting marker genes for the 10 distinct cell types. (f) Volcano plot of differentially expressed genes (DEGs) across the 10 distinct cell types. (g) Functional annotation of the 10 distinct cell types.

Through our single-cell analysis, we identified a total of 10 distinct cell types, including macrophages, T cells, hepatocytes, fibroblasts, NK cells, endothelial cells, monocytes, dendritic cells (DCs), plasma cells, and B cells, as shown in Figure 6a. Furthermore, the cellular composition of each sample is illustrated in Figure 6b. To gain further insights, we visualized the DEGs among the identified cell types, as depicted in Figure 6c. Moreover, UMAP projections of scRNA-seq data further classified these 10 distinct cell types, with representative marker genes, including macrophages (SLCO2B1), T cells (CD3D), hepatocytes (EPCAM), fibroblasts (COL1A1), NK cells (IFNG), endothelial cells (PECAM1), monocytes (S100A12), DCs (CD1E), plasma cells (JCHAIN), and B cells (MS4A1), as shown in Figures 6d,e. Additionally, we presented a volcano plot to visualize the DEGs of each cell type, as shown in Figure 6f. Furthermore, we performed functional analyses on different cell types to illustrate their biological roles, as depicted in Figure 6g.

### 3.5. The Critical Role of T Cells in the TIL Pathway

**Figure 7:**
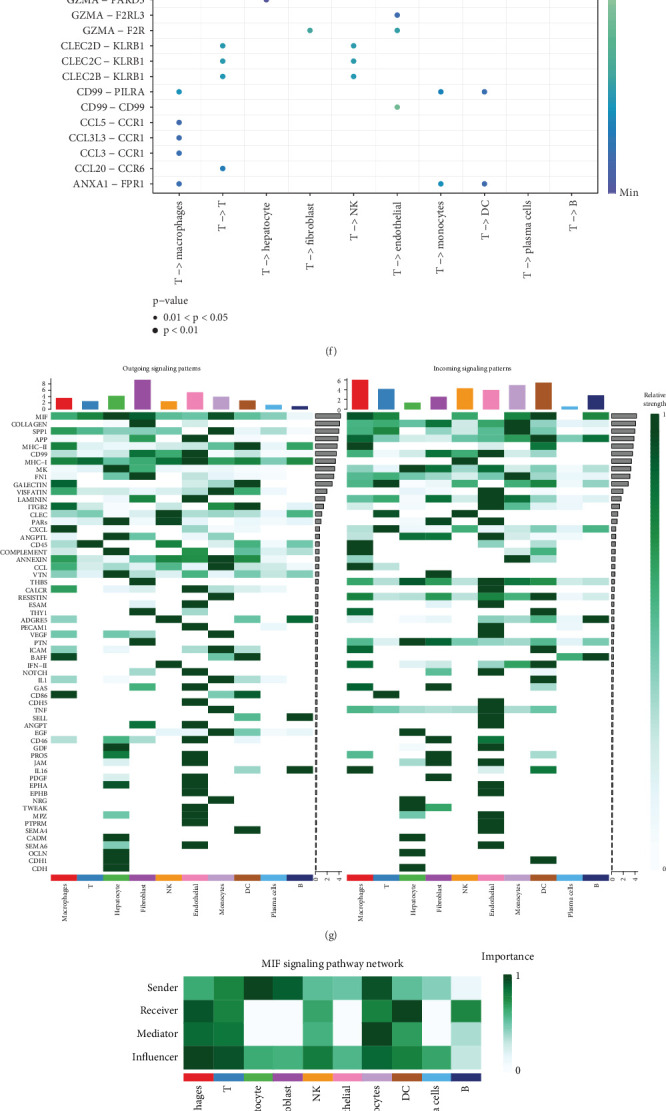
(a) UCell result for TIL in 10 distinct cell types. (b) UMAP projection highlighting TIL_UCell for the 10 distinct cell types. (c) Number of interactions and interaction weights among 10 distinct cell types. (d) Intercellular communication analysis for all pathways and MIF. (e, h, and i) MIF signaling pathway network among 10 distinct cell types. (f) Dotplot exhibiting significant interactions between T cell and other cell types. (g) Heatmap of outgoing signaling patterns and incoming signaling patterns in pathways. (j) Violin plot displaying MIF associated gene expression for the 10 distinct cell types. (k) Confirmation of MIF knockout in TALL-104 cells by Western blot analysis. (l, m) ERGIC3 expression in Hep 3B cells after coculture with different TALL-104 cell types by Western blot analysis. (n) Quantification of ERGIC3 expression from Western blot analysis. (o) MIF knockout in TALL-104 cells enhances cytotoxic effects on Hep 3B cells as assessed by CCK-8 assay. (p) Quantitative real-time PCR analysis of ERGIC3 mRNA expression in Hep 3B cells following coculture with different TALL-104 cell lines.

To determine which cell type the TIL pathway primarily functions in, we employed the UCell algorithm, which revealed high TIL expression in T cells, as shown in Figure 7a,b. Meanwhile, T cells were found to play a pivotal role in cellular communication, as illustrated in Figure 7c,d. Furthermore, these interactions predominantly involved immune cells and were mediated through the MIF signaling pathways (MIF-(CD47 + CXCR4) and MIF-(CD74 + CD44)), as depicted in Figures 7e, 7f, 7g, 7h, and 7i. Subsequently, MIF-associated genes were visualized across different cell types in Figure 7j.

### 3.6. The Impact of MIF on T-Cell Viability

Upon MIF knockout, T cells exhibited significantly enhanced cytotoxicity against Hep 3B cells compared to Mock-treated and normal T cells, which showed no statistically significant difference (*p* > 0.05 by two-tailed *t*-test) in Figure 7o. Furthermore, we assessed alterations in ERGIC3 expression within Hep 3B cells following coculture with distinct T-cell subsets. WB and qPCR analyses demonstrated that ERGIC3 expression was downregulated in Hep 3B cells upon T-cell exposure, paralleling the observed suppression of tumor cell proliferation in Figure 7m,p. Notably, MIF deficiency exacerbated this inhibitory effect, leading to further suppression of ERGIC3—a protumorigenic factor implicated in HCC progression.

## 4. Discussion

This study explores the immunosuppressive effects of TILs and their associated genetic characteristics in NASH-HCC using various bioinformatics and machine learning approaches. NASH is characterized by the presence of > 5% hepatic steatosis plus evidence of hepatocellular injury and inflammation, with or without fibrosis [20]. In the Western world, NASH-HCC currently represents 20% of all HCC cases and is expected to emerge as the leading cause of HCC worldwide by 2030 [21]. The development of NASH-HCC is linked to the immune microenvironment and reduced responsiveness to immunotherapy. In the context of NASH, both adaptive and innate immune cells have been shown to influence the liver microenvironment, driving the transition to NASH-HCC. These include CD4+ T cells and activated CD8+ T cells [22, 23]. However, T cells also play an antitumorigenic role. In a murine model of NASH-driven HCC, the removal of CD4+ T cells facilitated tumor progression [24]. The depletion of CD4+ cells significantly reduced the effectiveness of immune-based therapies in murine HCC models, suggesting that pre-existing liver disease may influence the response to immunotherapy [25]. First, GSEA and WGCNA were employed to analyze immune functions and identify gene modules highly associated with the TIL pathway. Subsequently, multiple machine learning methods were used to construct and optimize a TIL-related prognostic model, which was validated across multiple datasets, demonstrating strong predictive performance. Additionally, scRNA-seq analysis revealed the roles of different cell types in liver cancer, identifying T cells as key players in the TIL pathway, primarily mediating intercellular communication via the MIF signaling pathway. These findings provide new insights into the immune mechanisms of NASH-HCC and potential therapeutic strategies.

Our scRNA-seq analysis revealed that T cells play a pivotal role in the TIpathway within the NASH-HCC microenvironment. T cells serve as key mediators of antitumor immunity by recognizing and eliminating malignant cells. However, in NASH-HCC, their function is often suppressed due to an immunosuppressive TIME, which is characterized by chronic inflammation, regulatory T cell (Treg) infiltration, and increased expression of immune checkpoint molecules such as PD-1 and CTLA-4[26, 27]. This suppression compromises the ability of T cells to mount an effective immune response, allowing tumor cells to evade immune surveillance and proliferate.

Our study also identified that T cells engage in extensive intercellular communication, predominantly through the macrophage migration inhibitory factor (MIF) signaling pathway. Specifically, the MIF-(CD47 + CXCR4) and MIF-(CD74 + CD44) interactions were found to be highly active in T cells, suggesting that MIF signaling may contribute to the regulation of T cell function within the NASH-HCC microenvironment. MIF is a proinflammatory cytokine encoded by a genetically polymorphic locus. Initially identified as a cytokine produced by activated T cells, it is now recognized as a multifunctional key regulator secreted by various cell types involved in immune responses and physiological processes [28, 29]. MIF plays a crucial role in cell proliferation, tumorigenesis, and metastasis. It has been found to be overexpressed in various tumor types, including breast cancer, genitourinary cancers [30], melanoma [31], colorectal and head and neck cancers [32] and brain cancer [33]. However, MIF remains a multifaceted and controversial cytokine, as its role under pathological conditions appears to be diverse, exhibiting both protective and detrimental effects [34]. In Parkinson's disease, MIF suppresses inflammation and apoptosis while promoting autophagosome formation. The regulation of inflammatory responses is driven by an increase in the anti-inflammatory cytokine IL-10 and a reduction in the pro-inflammatory cytokine TNF-*α*. Furthermore, MIF significantly inhibits the cleavage of poly (ADP-ribose) polymerase (PARP), downregulates the expression of the pro-apoptotic gene Bax, and enhances mitochondrial membrane potential to prevent apoptosis [35]. This statement indicates that MIF (Macrophage Migration Inhibitory Factor) is involved in pro-inflammatory processes, as its increased expression in cancer cells and the stroma of breast cancer tissues suggests a role in tumor progression. The suggestion of MIF as a prognostic marker for breast cancer highlights its potential involvement in the inflammatory tumor microenvironment, which is often linked to tumor development and progression [36]. The interaction between MIF and its functional receptor CD74 activates the Akt and AMPK proteins through phosphorylation, enhances the MAPK signaling pathway that is involved in tumorigenesis, and downregulates the JNK mediated cell death [37, 38]. This study evaluates the role of MIF in liver cancer from a unique perspective based on TIL-related genes. Elevated MIF expression is often associated with decreased infiltration and impaired activity of CD8^+^ T cells, suggesting a negative regulatory role of MIF in TIL-mediated anti-tumor immunity [39]. As shown in Figure 6g, the potential biological processes mediated by MIF in liver cancer are revealed, with RNA splicing notably highlighted. Mutations in the MIF gene and epigenetic modifications in key gene promoters or enhancer regions may lead to abnormal expression or activation of proteins involved in signaling pathways that trigger malignant cell transformation, potentially serving as a crucial mechanism in MIF-driven liver cancer progression [40]. To more comprehensively reflect the complexity of the TIME, it is indeed essential to highlight the roles of other immune cells such as Tregs and MDSCs in the TIL pathway. Tregs are known to secrete immunosuppressive cytokines, such as IL-10, IL-35, and TGF-*β*, and can further suppress effective immune responses through mechanisms including nutrient depletion, IL-2 consumption, and cytolysis [41]. Complementary studies have specifically highlighted the roles of IL-10 and IL-35 in promoting BLIMP1-dependent inhibition of CD8^+^ TILs, underscoring the immunosuppressive influence of Tregs within the TIME [42]. PMN-MDSCs promote CTL apoptosis primarily via the Fas/FasL signaling pathway, while M-MDSCs exert immunosuppressive effects by producing nitric oxide, which dampens immune activation [43]. Additionally, M-MDSCs have the capacity to differentiate into immunosuppressive macrophages, further contributing to T cell inhibition [44].

In summary, our findings underscore the central role of T cells in the TIL pathway within NASH-HCC and highlight potential mechanisms contributing to their dysfunction. By elucidating the immunosuppressive factors affecting T cells, we provide insights that could inform the development of novel therapeutic strategies aimed at restoring effective antitumor immunity in NASH-HCC patients. These genes could potentially serve as the basis for novel diagnostic tools that enable more precise stratification of HCC patients according to their TIME profiles. For instance, TIL gene signatures may be incorporated into predictive models to guide immunotherapy decision-making, identifying patients who are more likely to benefit from ICIs or combination therapies. With further validation, these genes may also aid in monitoring treatment responses or disease progression, contributing to the advancement of personalized medicine in liver cancer.

## Data Availability

The data that support the findings of this study are available in NCBI Gene Expression Omnibus and The Cancer Genome Atlas at https://www.ncbi.nlm.nih.gov/geo/ and https://portal.gdc.cancer.gov/. These data were derived from the following resources available in the following public domain: GSE164760, https://www.ncbi.nlm.nih.gov/geo/query/acc.cgi?acc=GSE164760; GSE14520, https://www.ncbi.nlm.nih.gov/geo/query/acc.cgi?acc=gse14520; GSE76427, https://www.ncbi.nlm.nih.gov/geo/query/acc.cgi?acc=GSE76427; GSE149614, https://www.ncbi.nlm.nih.gov/geo/query/acc.cgi?acc=GSE149614; TCGA-LIHC, https://portal.gdc.cancer.gov/projects/TCGA-LIHC.

## Supporting Information

Supporting Information 1 Figure S1: (a) The expression levels of ERGIC3 in different clinical characteristics groups. (b) The expression levels of ERGIC3 in cancerous and adjacent normal tissues were compared. (c) The expression levels of PGF in different clinical characteristics groups. (d) The expression levels of PGF in cancerous and adjacent normal tissues were compared. (e) The expression levels of RHEB in different clinical characteristics groups. (f) The expression levels of RHEB in cancerous and adjacent normal tissues were compared.

Supporting Information 2 Figure S2 (a–c) GO enrichment result in high ERGIC3 and low ERGIC3 group. (d) Circlize plot of GO enrichment result in high ERGIC3 and low ERGIC3 group. (e) GSEA result in high ERGIC3 and low ERGIC3 group.

Supporting Information 3 Table S1: Scale-free topology fitting index related data. Table S1 provides the scale-free topology fitting index data used in the analysis and demonstrates the rationale behind our parameter selection.
