# Supplementary figures and images for: Exploration of the Roles and Mechanisms Between Tumor-Infiltrating Lymphocytes and Hepatocellular Carcinoma Based on Single-Cell Transcriptomics
Source: Int J Genomics. 2025 Aug 20;2025:1575734. doi: 10.1155/ijog/1575734 (PMC12390586; doi:10.1155/ijog/1575734)

A

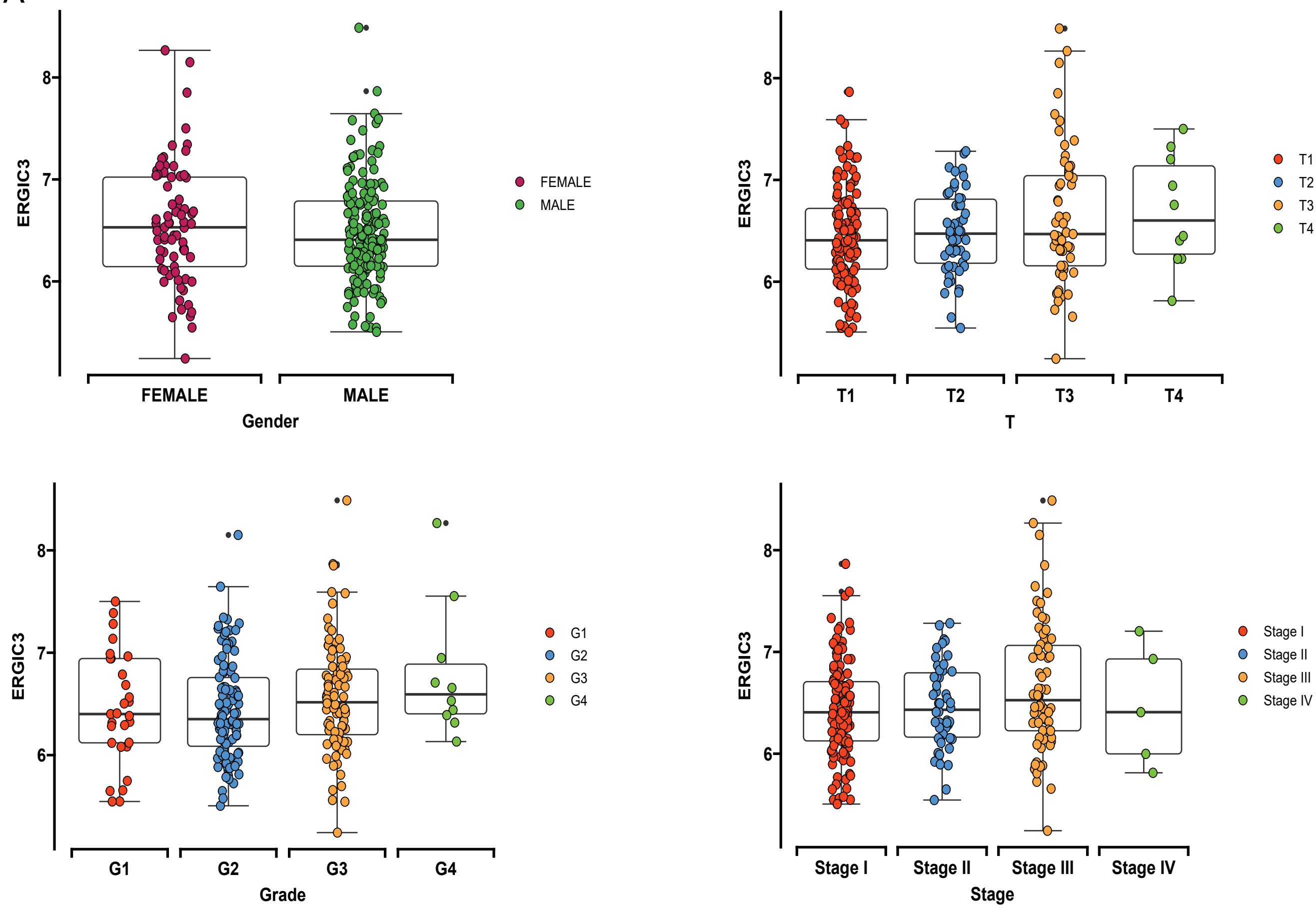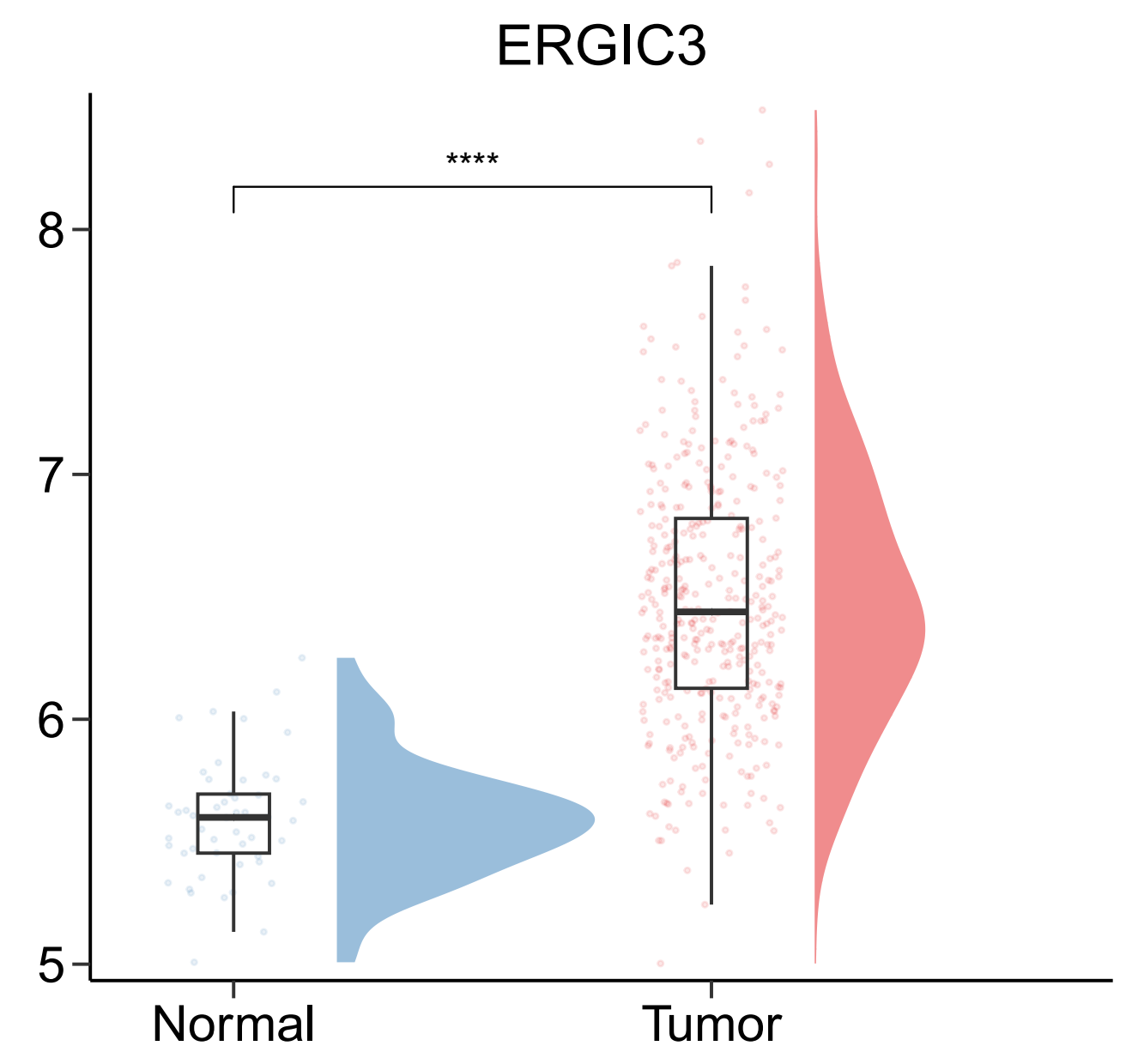

B

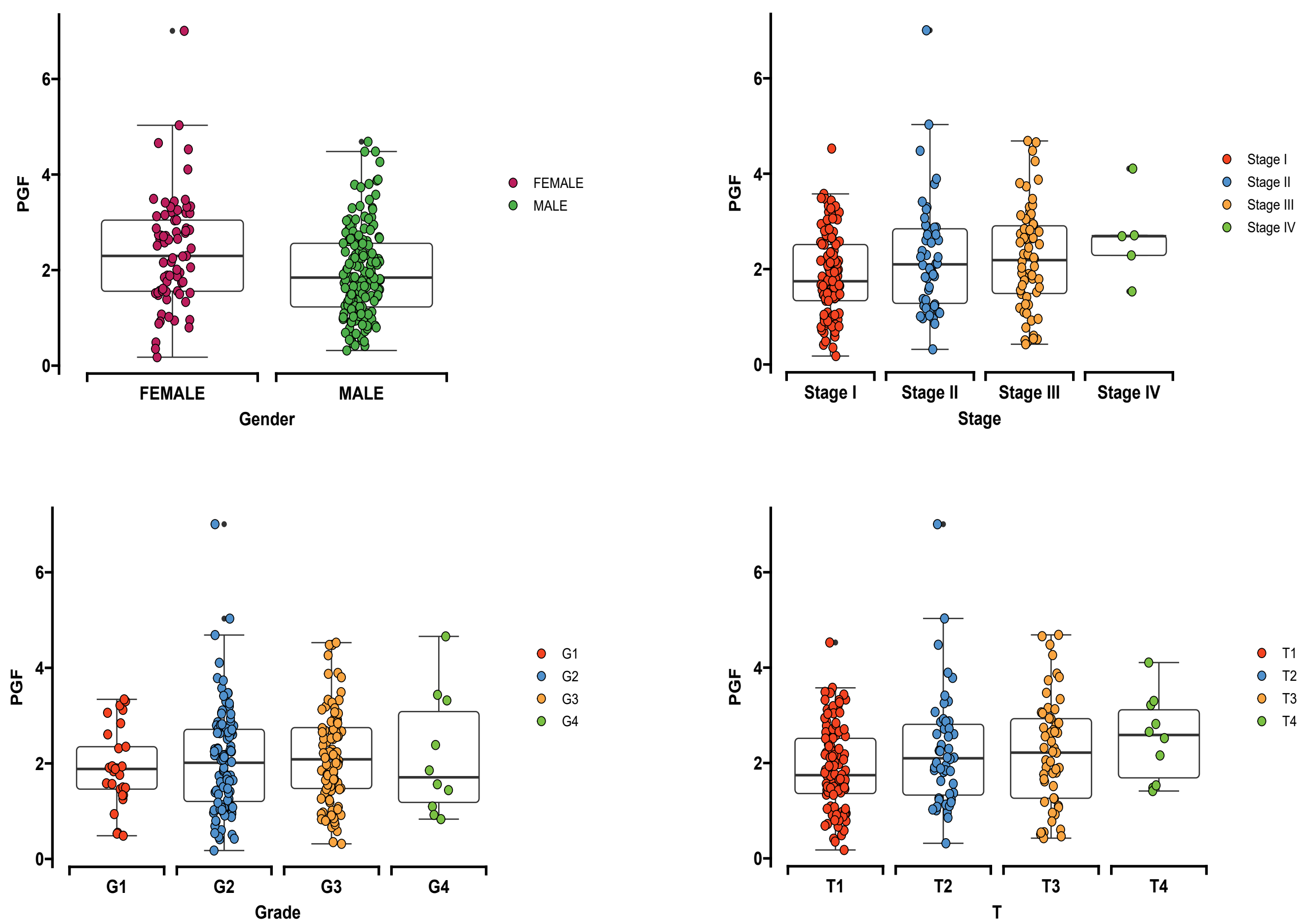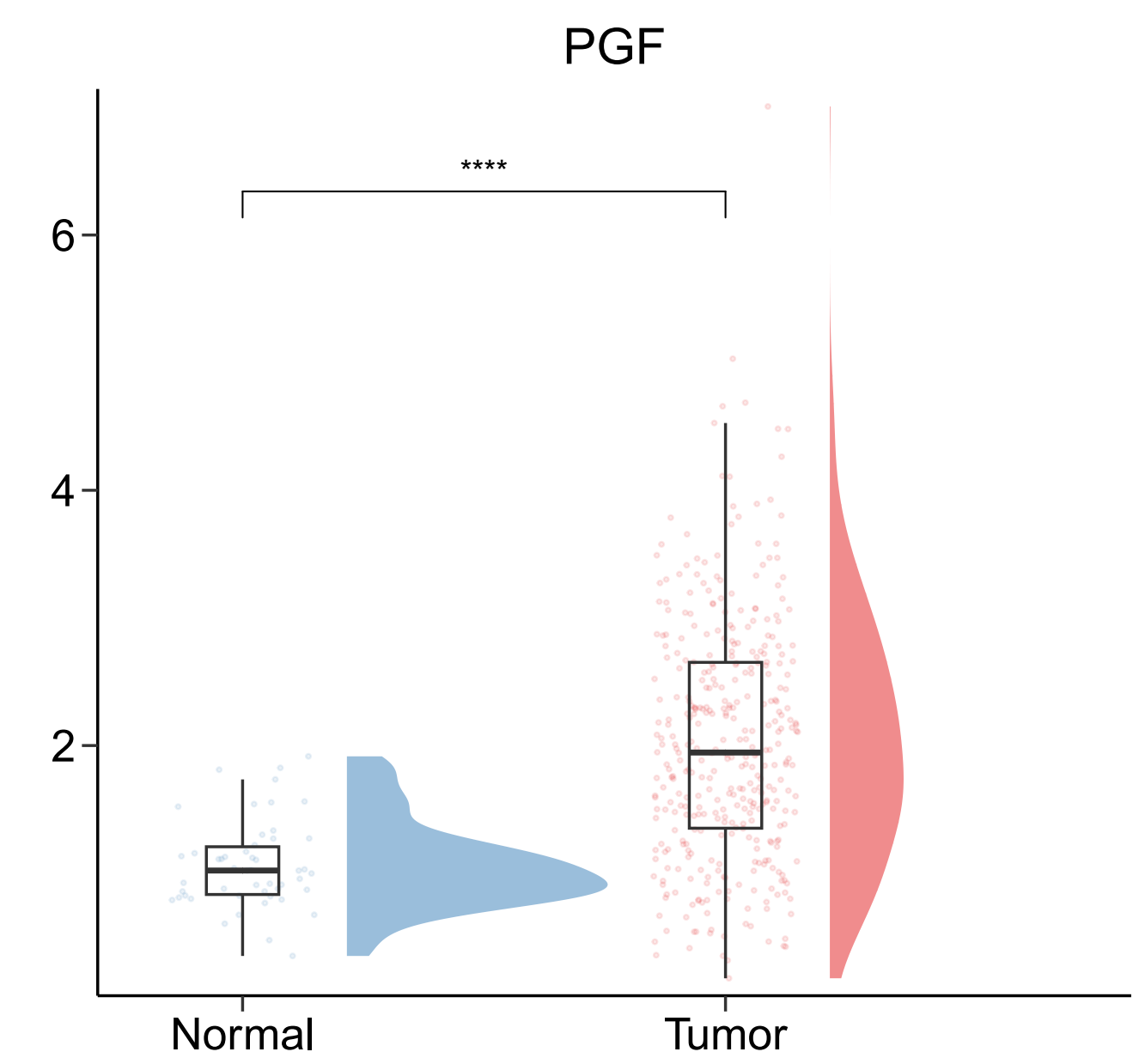

C

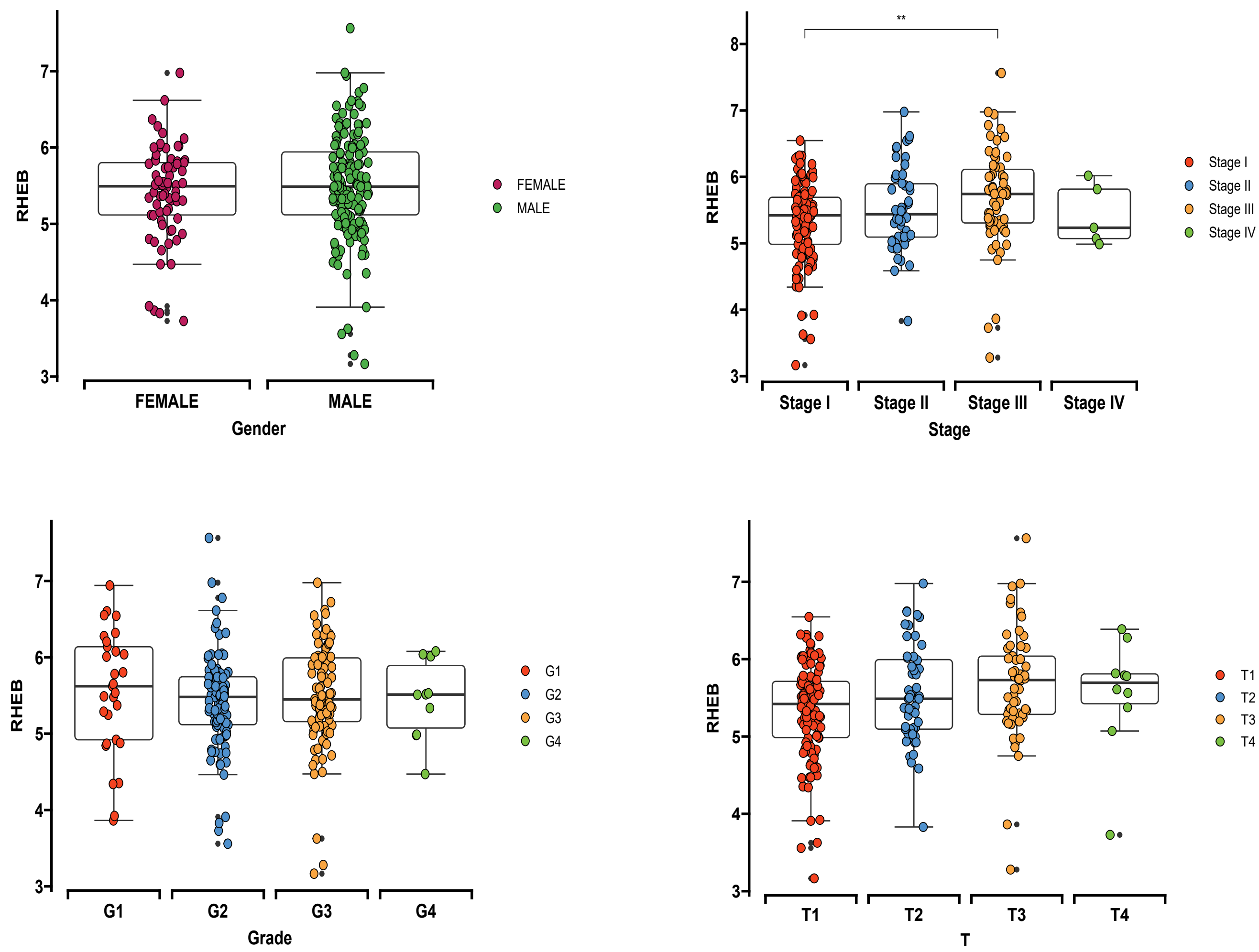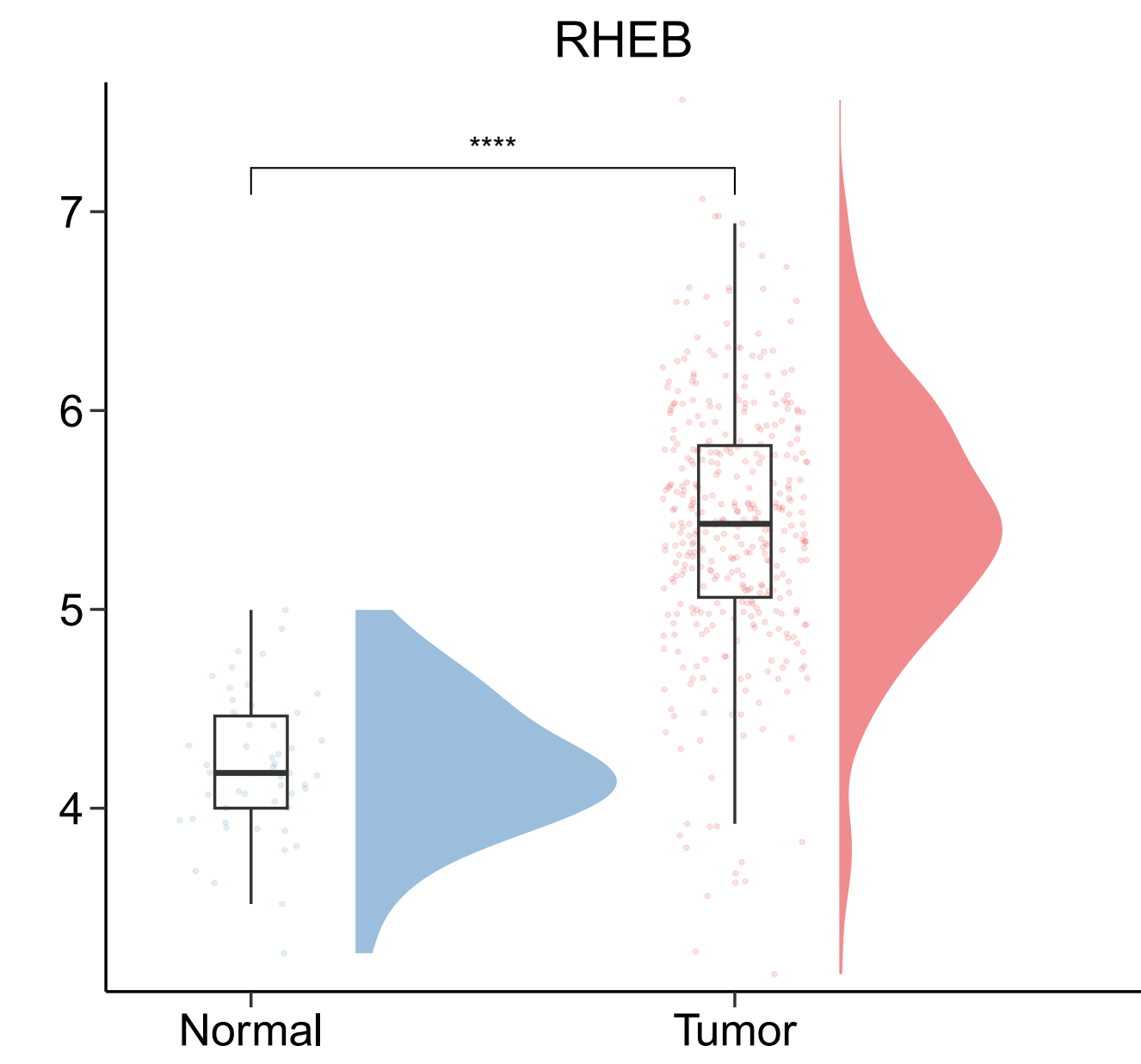

Supplement: Supporting Information 1 — Figure S1: (a) The expression levels of ERGIC3 in different clinical characteristics groups. (b) The expression levels of ERGIC3 in cancerous and adjacent normal tissues were compared. (c) The expression levels of PGF in different clinical characteristics groups. (d) The expression levels of PGF in cancerous and adjacent normal tissues were compared. (e) The expression levels of RHEB in different clinical characteristics groups. (f) The expression levels of RHEB in cancerous and adjacent normal tissues were compared. [file 1575734.f1.pdf]

Term

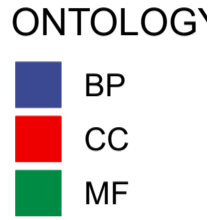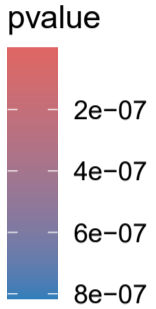

102

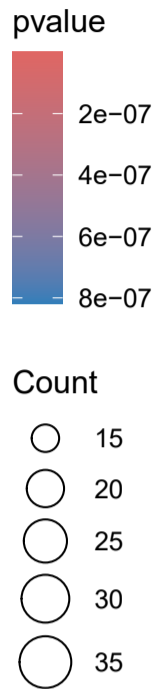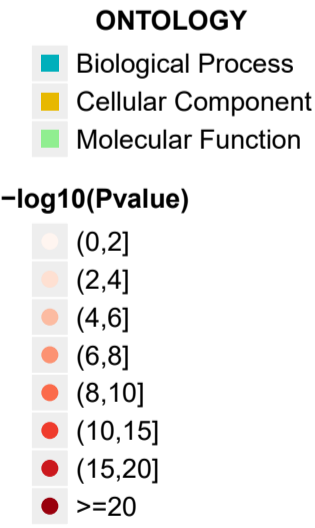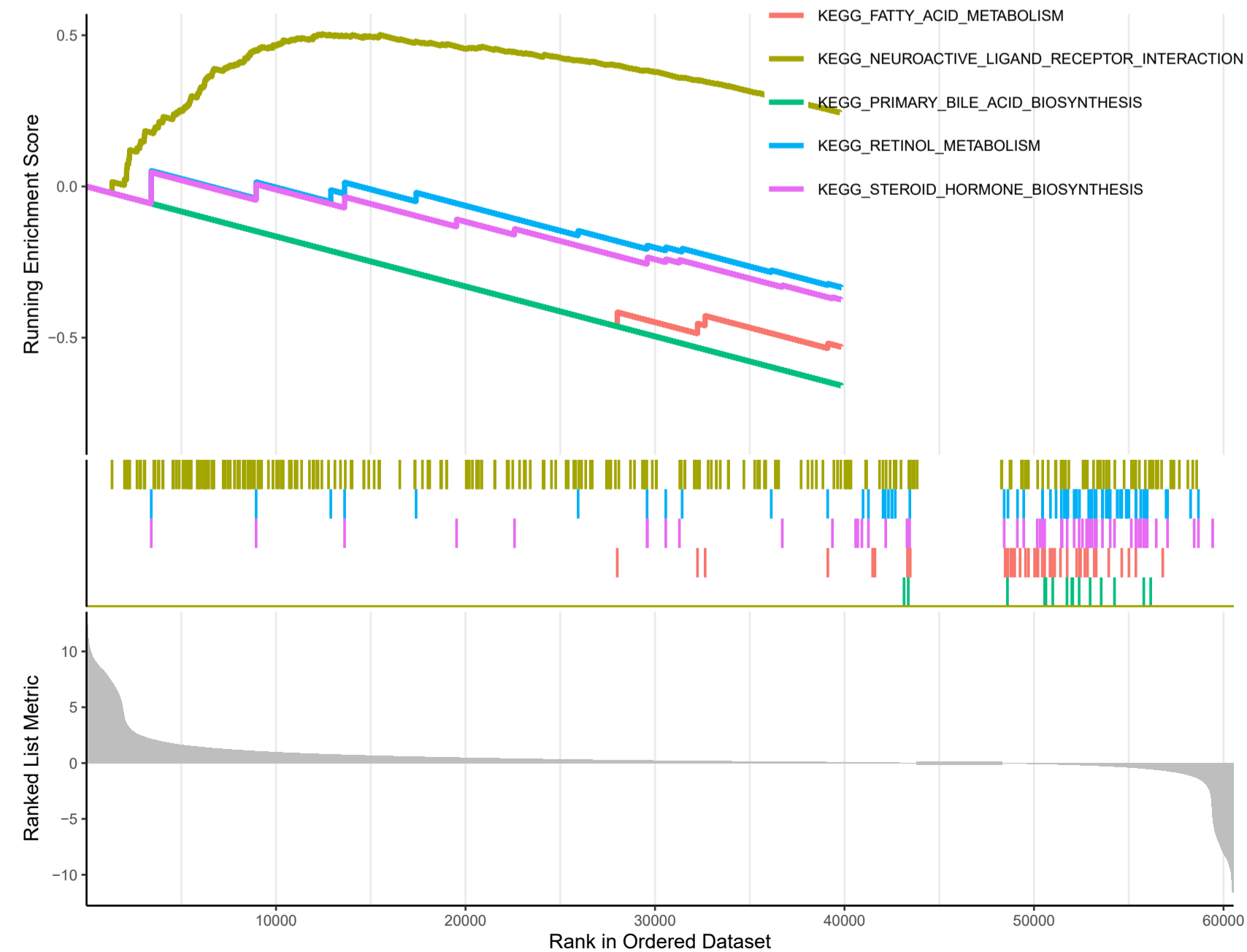

Supplement: Supporting Information 2 — Figure S2 (a–c) GO enrichment result in high ERGIC3 and low ERGIC3 group. (d) Circlize plot of GO enrichment result in high ERGIC3 and low ERGIC3 group. (e) GSEA result in high ERGIC3 and low ERGIC3 group. [file 1575734.f2.pdf]
